# Metagenomic insight into the diversity and biogeochemical functions of microbial communities in the maar tropical Lake Atexcac

**DOI:** 10.1099/mic.0.001714

**Published:** 2026-06-04

**Authors:** Rodrigo Hernández-Velázquez, Justo Salvador Hernández-Avilés

**Affiliations:** 1 Laboratory of Limnoecology, Biology Department, UMIEZ, FES Zaragoza, National Autonomous University of Mexico (UNAM), Mexico City, Mexico

**Keywords:** maar lake, metagenome, carbon cycle, nitrogen cycle, sulphur cycle

## Abstract

Warm monomictic maar lakes in tropical regions represent dynamic systems where thermal stratification generates strong vertical gradients in oxygen availability and redox conditions, shaping microbial community structure and function. Lake Atexcac (Puebla, Mexico) undergoes seasonal stratification and episodic whiting events that provide a framework to examine microbial responses to changing hydrodynamic conditions. In this study, we applied deep shotgun metagenomic sequencing to characterize the taxonomic composition and functional potential of microbial communities across the epilimnion, metalimnion and hypolimnion during two contrasting stratification phases: early stratification associated with a whiting event and a later, well-established stratification period.

Metagenomic profiles revealed a clear vertical organization of microbial communities, with samples clustering primarily according to thermal strata and the metalimnion displaying the highest genetic differentiation. Genome-resolved analyses enabled the recovery of a large number of metagenome-assembled genomes, with marked differences in their vertical distribution between hydrodynamic phases. The recovered genomes encompassed diverse metabolic pathways related to carbon, nitrogen and sulphur transformations, reflecting the heterogeneous redox conditions along the water column. Notably, sulphur-related metabolisms were widespread across strata, and *Chlorobiota*-affiliated genomes and metagenomic reads were consistently detected in suboxic layers. These organisms were found to harbour diverse thiosulphate disproportionation pathways and are thought to play an important role in the sulphur cycle that has not previously been reported in this type of lacustrine system.

Overall, this study provides a genome-resolved perspective on microbial diversity and metabolic potential in a stratified tropical maar lake and establishes a baseline for future comparative and process-oriented studies integrating water column and sediment microbial communities.

## Data Availability

Sequences can be fetched through NCBI using the PRJNA1249960 BioProject number. BioSample accessions are SAMN47925824, SAMN47925825, SAMN47925826, SAMN47925827, SAMN47925828 and SAMN47925829 (https://www.ncbi.nlm.nih.gov/bioproject/?term=PRJNA1249960).

## Introduction

Volcanic lakes, due to their origin, geomorphology and thermal characteristics, provide a valuable opportunity to study microbial assemblages involved in various biogeochemical cycles, including carbon, nitrogen, phosphorus, silica and sulphur. In particular, the warm monomictic maar lakes in tropical regions exhibit a prolonged stratification lasting around 8 months [1,2]. In these systems, varying thermal conditions throughout the water column create contrasting levels of gas concentrations, especially for dissolved oxygen. Consequently, the water column becomes vertically structured into an oxygen-rich epilimnion, a metalimnetic zone with varying gradients of oxygen and anoxic conditions in the hypolimnion [3]. These conditions, in turn, influence different redox potentials related to the chemical states of elements and their transformations. Notably, stratified lakes with oxygen deficits are of particular interest, as they may be significant sources of greenhouse gases and hotspots within the carbon cycle [4].

The whiting event occurring in some of these lakes enables these systems to function as potential carbon sinks, and this phenomenon is characterized by a turquoise colouration of the water surface caused by the suspension of fine calcium carbonate (calcite) particles that scatter light [5,6]. These carbonates can precipitate in significant amounts, increasing the concentration of dissolved inorganic carbon at the bottom of the lakes [7].

Whiting events have been documented in various lake systems, including the Great Lakes in North America [8] and several hardwater lakes in Europe [9], as well as the tropical Lake Kivu [10]. Whiting events are primarily linked to the carbonate nucleation process, which occurs when picocyanobacteria utilize bicarbonates for photosynthesis [7]. Additionally, other bacterial metabolic routes, such as ureolysis, denitrification, ammonification, sulphate reduction and methane oxidation, are also involved [11]. The whiting event occurs annually during thermal stratification in Atexcac, a maar lake located in a semi-arid tropical region of the Oriental watershed in Puebla. This lake is characterized by hyposaline, alkaline and hard water conditions that promote this event [2].

The study of microbial diversity in aquatic ecosystems using metagenomic approaches has advanced substantially in recent years, particularly in freshwater lakes at temperate latitudes. These systems have received considerable attention because of their high microbial diversity, a central role in biogeochemical cycling, and pronounced sensitivity to climate change [12]. Collectively, these advances demonstrate that large-scale metagenomic surveys of freshwater lakes provide an unprecedented resolution of microbial diversity and functional potential across trophic gradients. Continental-scale efforts, such as the study of 308 Canadian lakes, highlight the power of metagenome-assembled genomes (MAGs) to link microbial community structure with biogeochemical processes, establishing a robust framework for understanding ecosystem functioning and predicting freshwater responses to ongoing environmental change [13]. Long-term metagenomic time series from Lake Mendota demonstrate that freshwater microbial communities exhibit pronounced seasonal and decadal dynamics, with recurrent strain-level shifts driven by environmental variability, highlighting the tight coupling between microbial ecology, evolution and ecosystem function [14]. Other studies conducted in lakes and ponds in boreal and subarctic regions, which show stratification and a deficit of oxygen in the hypolimnion, generated a comprehensive dataset of 267 shotgun metagenomes [4]. Research on tropical lakes emphasizes understanding microbial communities and their roles in biogeochemical cycles across different depths. In Lake Tanganyika, the deepest and largest tropical freshwater lake, MAGs associated with nitrogen and sulphur cycles were found to be abundant [15]. Other studies have characterized the microbial diversity and metabolic functions during mixing and stratification periods in large deep reservoirs using metagenomic sequencing and quantitative PCR (qPCR) [16] and warm monomictic lakes such as Lake Kinneret through metagenomics [17]. Warm monomictic lakes have been understudied, with prior work often limited to qPCR-based quantification of selected functional genes (e.g. nitrogen-cycle genes) [18], where diversity and structure of prokaryotic communities in water, microbialites and sediments have been assessed through 16S rRNA-based microbial diversity analyses [19]. Additionally, studies on microbial diversity and nitrogen metabolism in Lake Yojoa, Honduras, have utilized metatranscriptomics [20]. Despite these recent studies, the sulphur cycle and other relevant cycles have not been characterized in detail.

In tropical lakes, the dynamics of biogeochemical processes change continuously throughout the year, primarily influenced by periods of stratification [21]. Picocyanobacteria play a significant role in photosynthesis in the epilimnetic and metalimnetic layers, along with other prokaryotic functional groups, that exhibit high metabolic potential, especially in the warm hypolimnetic waters found at low latitudes [19,22]. Maar lakes with monomixis are characterized by stratification, in which gradual changes in water density occur due to temperature variations within the thermocline. These temperature differences affect the depth, amplitude and intensity of the gradient, subsequently impacting the stratification of the entire water column over time [1]. This process leads to early stratification in spring, a well-established state in summer and late stratification in autumn [3]. The dynamic nature of these lakes makes it imperative to characterize multiple periods throughout the water column.

The present study assesses the composition of the microbial community and its metabolic contributions to key biogeochemical processes using a metagenomic approach. Focusing on the first two hydrodynamic stratification periods of Lake Atexcac, we examine consecutive yet contrasting conditions characterized by a whiting event and by the development of pronounced thermal and chemical gradients in the water column. We hypothesize that these stratification-driven redox gradients vertically structure microbial communities and their functional gene repertoires, promoting sulphur-based and heterotrophic carbon metabolisms, favouring nitrogen reduction pathways and limiting the development of methanogenic and anaerobic ammonium oxidation (anammox)-related metabolisms within the water column. By providing the first metagenomic comparison of microbial community structure and metabolic potential across successive stratification phases in a tropical monomictic lake, this study offers novel insights into microbial controls on carbon, nitrogen and sulphur cycling.

## Methods

### Sampling site

**Fig. 1.**
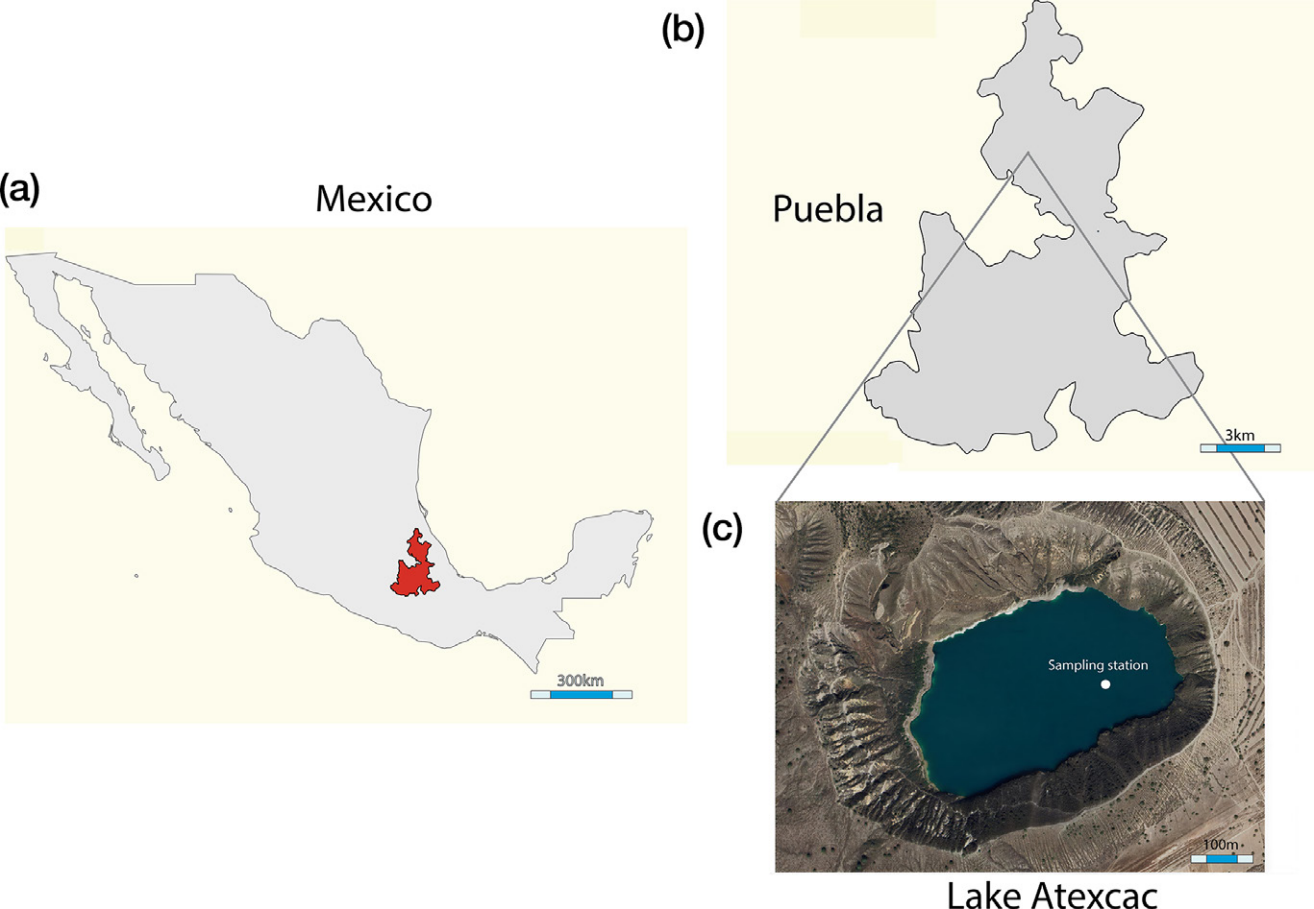
Location of the study area and sampling site. (**a**) Geographic location of the state of Puebla in Mexico. (**b**) State of Puebla indicating the municipality of Guadalupe Victoria, where Lake Atexcac is located. (**c**) Lake Atexcac, based on a Google Maps image, with the main pelagic sampling station (white dot) located at the deepest point of the basin. This station represents pelagic conditions and encompasses the full water column, including the epi-, meta- and hypolimnion of this warm monomictic maar lake.

Atexcac is a maar lake in central Mexico (19° 19′ 50.68″–19° 20′ 12.30″ N and 97° 26′ 45.99″–97° 27′ 15.37″ W; 2,510 m a.s.l.) (Fig. 1). It is elliptical in shape, with abrupt slopes, a surface area of 0.31 km², and a maximum depth of 34 m. The lake is hyposaline (6.6 g l⁻¹) and alkaline (pH 8.2–9.3) and is dominated by sodium–magnesium and chloride bicarbonate and carbonate ions [2]. Atexcac is a warm monomictic lake that has undergone a trophic shift from oligotrophic to mesotrophic conditions [23]. The regional climate is classified as temperate semi-arid, with an average annual rainfall of 518 mm and a mean annual temperature of 14.8 °C [24].

### Measurements *in situ*

Sampling was conducted in the limnetic zone at the deepest part of the lake, located at 19° 20′ 2.04″ N 97° 26′ 59.49″ W (Fig. 1). Two sampling events took place: one in June 2022, coinciding with the early stratification stage when a whiting event was observed in the lake, and another in August 2022, under conditions of well-established stratification. Sampling depths for physicochemical measurements, prokaryoplankton characterization and DNA analysis included the near-surface and near-bottom layers, the euphotic zone, thermocline, oxycline and the deep chlorophyll maximum. During early stratification, coinciding with a whiting event in the lake (June 2022), samples were collected from seven depths: three within the epilimnion (5, 10 and 12 m), two in the metalimnion (13 and 16 m) and two in the hypolimnion (25 and 31 m). In contrast, during well-established stratification (August 2022), nine depths were sampled, with three depths representing each of the stratified layers epilimnion (5, 10 and 15 m), metalimnion (17, 19 and 21 m) and hypolimnion (25, 27 and 31 m). Vertical profiles of temperature, dissolved oxygen concentration, pH, electrical conductivity, oxidation–reduction potential (ORP) and chlorophyll a were recorded along the water column (1 m spatial resolution) with a Hydrolab MS5 water quality multiprobe (Hach, Loveland, CO, USA).

The Secchi disc depth (Z_SD_) was measured, and the euphotic depth (Z_eu_) was estimated by multiplying Z_SD_ by a factor of 3.0 [25]. Water samples were taken with a Van Dorn sampler at different depths for the evaluation of inorganic nutrients, sulphates and sulphides. Water samples were also fixed with 2% formalin for the analysis of autotrophic picoplankton.

Ex situ spectrophotometric methods were used to analyse nitrates, nitrites, soluble reactive phosphorus (orthophosphates), total phosphorus and sulphate concentrations after sample filtration through Whatman quantitative filter paper, ashless, grade 42 (APHA, 2016). Ammonia and sulphide ions were determined *in situ* with DR900 Multiparameter Portable Colorimeter (Hach Company, Loveland, CO, USA) with the Salicylate Method [0.02 to 2.50 mg l^−1^ NH_3_–N (LR)] and the USEPA Methylene Blue Method (0.01 to 0.70 mg l^−1^ S_2_^–^), respectively.

Autotrophic picoplankton (APP) were concentrated onto white polycarbonate membranes (0.2 µm) and counted by autofluorescence in an epifluorescence microscope (Leica, Germany). At each sampling depth, bacterial biomass (organic carbon) was estimated as follows: photographs of APP samples were taken with a digital colour camera, Canon S45 with the program Canon Utilities Zoom Browser 6.9. Between 10 and 15 images of each preparation were taken to measure ~1×10^3^ cells. The images were analysed by ImageJ (USA) software [26] with additional volume-approximating macros [27]. Organic carbon content was calculated according to the method previously described [28].

### DNA extraction and sequencing

The water samples for each layer were pooled and filtered using a 47 mm diameter polycarbonate membrane with different pore sizes, first 3 µm to remove larger plankton, then 0.8 µm and 0.22 µm to evaluate procarioplankton DNA. The 0.8 µm and 0.22 µm membranes were frozen (−80 °C) until the DNA extraction. DNA was extracted using the DNeasy PowerSoil Pro Kit (MoBio, Carlsbad, CA, USA). After DNA extraction for each depth, both size fractions were pooled according to the specific layer of thermal stratification for each sampling month (epilimnion, metalimnion and hypolimnion).

The DNA concentrations were quantified using Qubit 2.0 Fluorometer (Invitrogen, Thermo Fisher Scientific, Carlsbad, CA, USA), and its quality and concentration were verified. Library preparation and sequencing were conducted at the QB3 Genomics, UC Berkeley, Berkeley, CA. Libraries were prepared using the KAPA HyperPrep library preparation kit (Roche) with stub Y-adapters and unique dual indexing. Samples were sequenced to generate 150 bp paired-end reads on an Illumina NovaSeq 6000 platform (Illumina, San Diego, CA, USA), with a sequencing effort of ~100 million reads per sample.

### Metagenome assembly

Inspection of the read quality per metagenome was performed using FastQC v. 0.11.8 [29], and quality trimming was done using Trimmomatic v 0.39 [30] with the parameters LEADING and TRAILING set to 30. After verifying with FastQC [29] that per-base sequence quality was as expected, assembly was conducted with MEGAHIT 1.2.9 [31] using the default parameters.

### Binning

For binning, MaxBin 2.27 [32] was first used with the default parameters, using the trimmed paired reads for each sample as input for frequency estimation. In a similar way, MetaBAT 2.15 [33] was used, setting the minimum contig length to 1,500 and the minimum contig depth to 2 when creating the contig depth summary tables. To create the input abundance files for this program, the bbmap.sh (v. 38.18) script from the BBtools [34] suite was used to map the reads to the contigs obtained from the assembly for each sample, setting the deterministic flag to true. The BAM files were then sorted using Samtools 1.6 [35]. To obtain a consensus from both binning tools, DAS Tool [36] was run with the default settings. CheckM v1.2.1 [37] was used to estimate completeness and contamination per bin. Bins were then classified into high-quality and medium-quality MAGs based on the completeness and contamination criteria proposed by the MIMAG standards, with high-quality MAGs defined as having ≥90% completeness and ≤5% contamination and medium-quality MAGs defined as having ≥50% completeness and ≤10% contamination [38].

### Read-level metagenomic sketch and non-metric multidimensional scaling

Quality-trimmed metagenomic reads were summarized using MinHash sketching as implemented in MASH v2.1 [39]. For each metagenome, sketches were generated using a sketch size of 10,000,000 hashes, and pairwise between-sample distances were computed from these sketches to obtain a distance (dissimilarity) matrix describing read-level relatedness among metagenomes. This distance matrix was used as input for ordination by non-metric multidimensional scaling (NMDS) in vegan v2.1 [40] using the metaMDS function with k=2 dimensions and 50 maximum random starts (trymax=50). The final configuration was selected as the solution with the lowest stress after iterative optimization, and ordination quality was assessed using the stress value, a stress scree plot across 1–4 dimensions. NMDS coordinates were visualized in ggplot2 with samples coloured by hydrological phase and shaped by water-column layer.

### Read-level taxonomic assignment

The taxonomy of quality-trimmed reads was obtained using the Kaiju program (1.9.2), enabling the greedy mode and with remaining default settings. The RefSeq database (23 March 2022) provided online (https://bioinformatics-centre.github.io/kaiju) by the authors was used to classify the reads. Kaiju outputs were merged and converted to a phyloseq [41] table using the script kaiju2phyloseq.py from the Biopythonpieces GitHub repository (https://github.com/gisleDK/Biopythonpieces). Phyloseq (version 1.38.0) [41] was used to filter reads that could not be assigned at the phylum level, and phyla that comprised less than 0.0001 of the reads (i.e. <0.01% of the abundance) were also excluded.

### Biogeochemical cycle prediction

Prediction of the key enzymes involved in the main biogeochemical cycles – carbon, nitrogen and sulphur – was performed using the METABOLIC v.4.0 software [42]. The medium- and high-quality MAGs per sample were used as an input, and reads were also provided to estimate MAG coverage in each sample with the METABOLIC-G implementation [42]. This software also allows obtaining taxonomic classification of the MAGs through GTDB-Tk v0.1.3 [43] which uses the GTDB (R89) database, a measure of relative evolutionary divergence and average nucleotide identity [43,44].

## Results

### Assembly and MAG recovery

Assembly metrics and MAG recovery are summarized in Table 1. Among all samples, the hypolimnion during the whiting event yielded the longest assembly and the highest number of medium- and high-quality MAGs. In both hydrological phases, deeper layers generally yielded more MAGs than surface layers. The two longest contigs were obtained from the hypolimnion and epilimnion samples during the whiting event. Overall, 325 (whiting event) and 275 (stratification) medium- and high-quality MAGs were recovered.

**Table 1.** Assembly statistics for each sample and number of high- and medium-quality MAGs

	Total assembly length	Mean contig length	L50	N50	Longest contig	High-quality MAGs	Medium-quality MAGs
Epilimnion whiting event (early stratification)	622,938,632	1,214.74	3,210	22,919	827,759	46	27
Metalimnion whiting event (early stratification)	747,989,515	1,278.09	3,566	25,070	586,743	51	44
Hypolimnion whiting event (early stratification)	1,307,726,813	1,245.44	2,895	56,763	930,158	74	61
Epilimnion well-established stratification	772,365,494	1,268.2	3,680	27,460	598,488	46	39
Metalimnion well-established stratification	1,118,453,088	1,210.96	2,742	55,803	675,861	63	50
Hypolimnion well-established stratification	1,113,982,052	1,127.52	2,129	74,377	582,321	45	53

### Biogeochemical cycles of carbon, nitrogen and sulphur

**Fig. 2.**
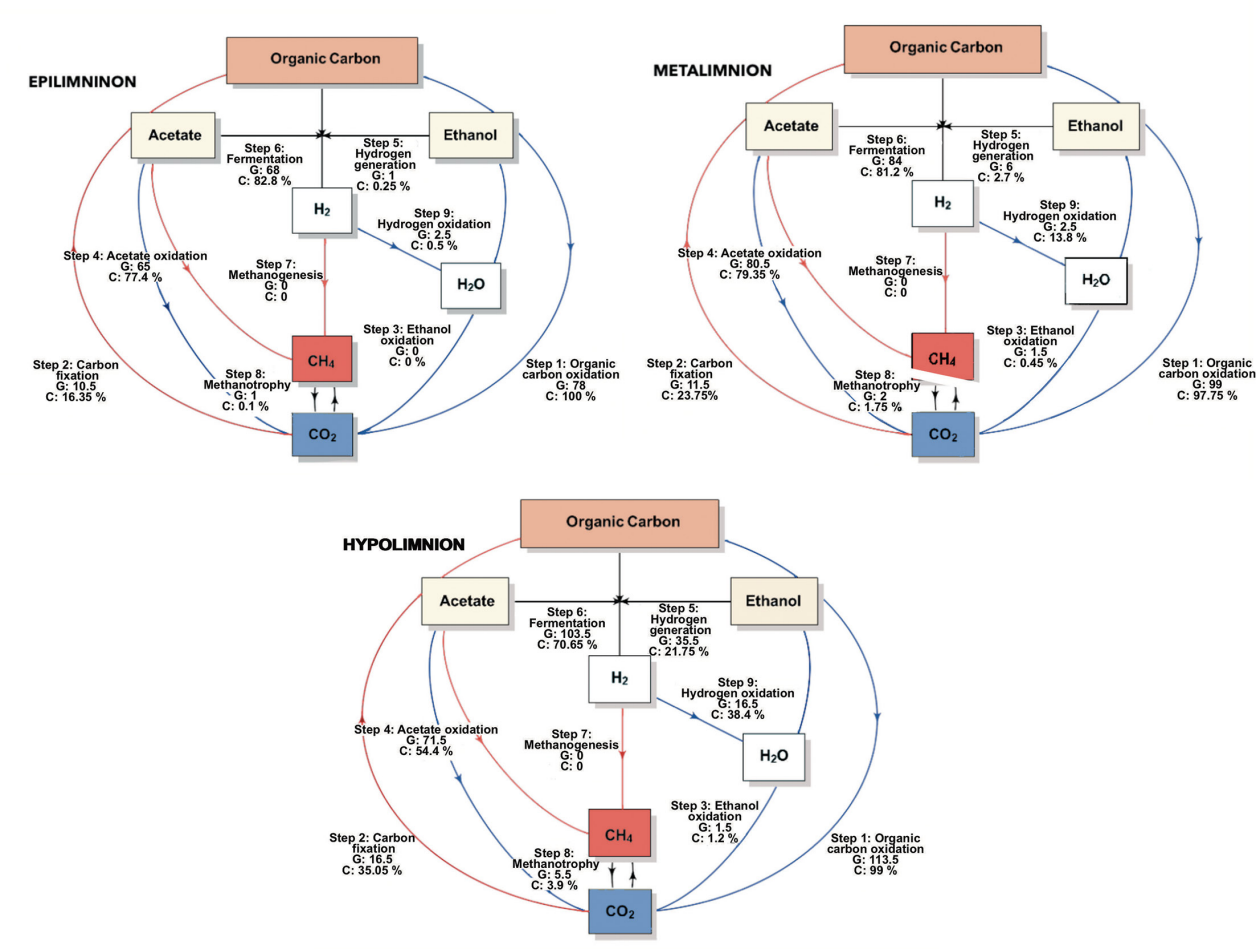
Mean number of genomes (G) and mean coverage (%) (C) of carbon cycle–related metabolic processes across water-column layers, averaged over early and well-established stratification periods. Red lines indicate reduction processes, whereas blue lines indicate oxidation processes.

**Fig. 3.**
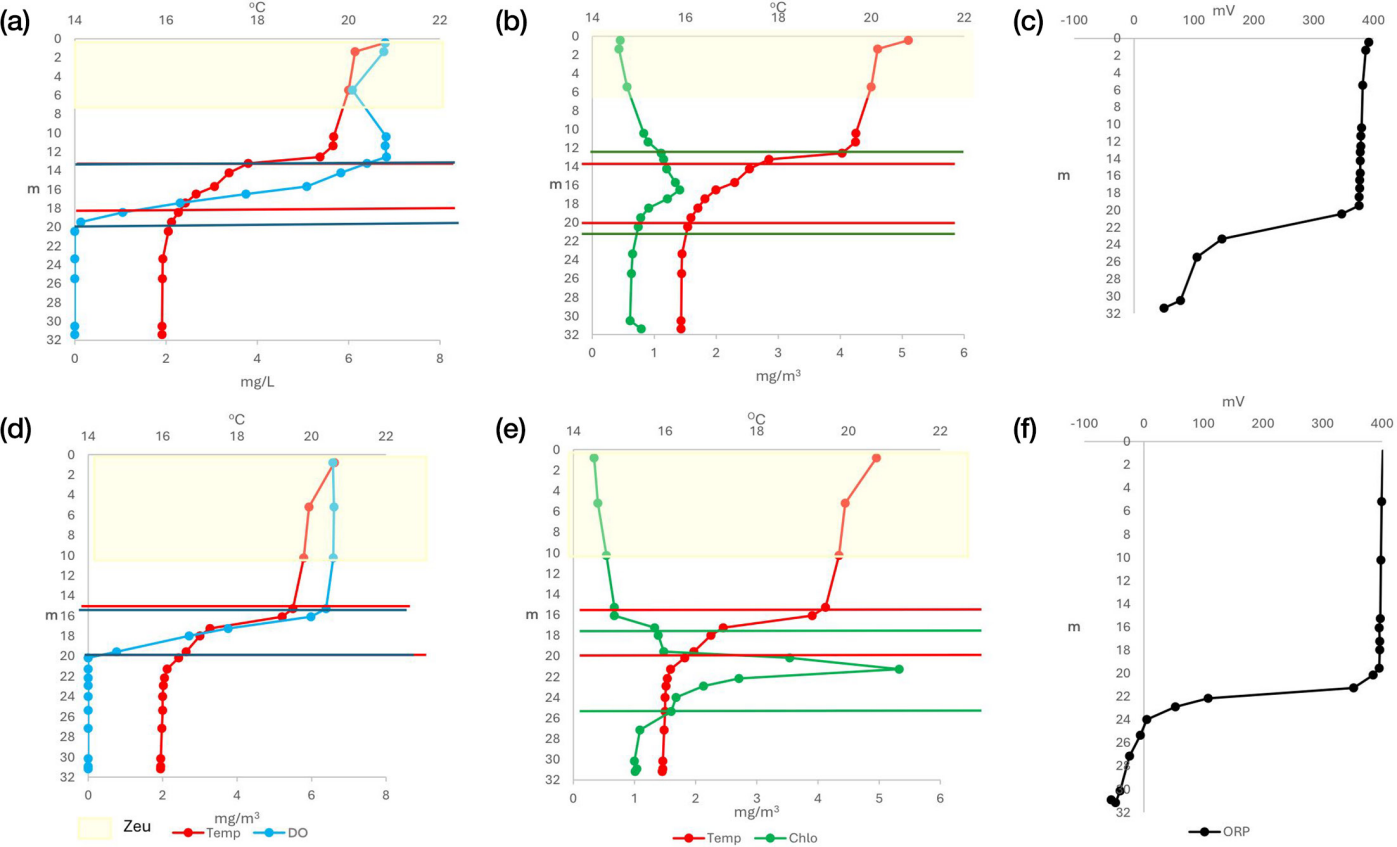
Vertical profiles measured during two stratification stages: early stratification (**a**) and well-established stratification (**b**). Panels a.1 and b.1 show temperature (Temp) and dissolved oxygen (D0) profiles; panels a.2 and b.2 show temperature and chlorophyll a (Chlo) profiles; panels a.3 and b.3 show ORP profiles. Panels a.1–a.3 correspond to early stratification, whereas panels b.1–b.3 correspond to well-established stratification. The yellow shaded area indicates the euphotic depth (Zeu). Horizontal red lines indicate the thermocline, blue lines indicate the oxycline, and green lines indicate the deep chlorophyll a maxima.

Carbon fixation–related genomes and gene coverages were detected throughout the water column of Lake Atexcac, increasing towards the metalimnion and hypolimnion (Fig. 2). Their vertical distribution coincided with the oxycline and the anoxic hypolimnion below 20 m depth (Fig. 3a.1, b.1), reflecting the close coupling between carbon assimilation and environmental gradients such as light availability, redox conditions, the development of deep chlorophyll maxima (DCM) and picocyanobacterial activity in this alkaline system. During June, coinciding with the whiting event, light penetration was limited to the upper 6 m, resulting in chlorophyll a concentrations below 1 mg m⁻³ in both the epilimnion and hypolimnion, while a DCM of 1.41 mg m⁻³ developed in the metalimnion (Fig. 3a). This peak corresponded to a bloom of picocyanobacteria *Synechococcus* (Fig. S1A, available in the online Supplementary Material). By August, following bloom decline, light penetration increased to 11 m, chlorophyll a concentrations reached ~1 mg m⁻³ in the epilimnion and hypolimnion, and a more pronounced metalimnetic DCM developed, reaching 5.33 mg m⁻³ (Fig. 3b.2), concurrent with increased picocyanobacterial abundance (Fig. S1B). At the same time, redox potential decreased with depth, reaching negative values at 26 m in August (Fig. 3a.3, b.3), indicating a transition towards reducing conditions that may favour non-phototrophic carbon fixation pathways in deeper layers.

Organic carbon oxidation was the dominant carbon-related process based on both genome counts and coverage, increasing with depth and with the establishment of thermal stratification (Figs2  3a.1, b.1). Acetate oxidation was well represented across all water-column strata and yielded carbon dioxide as the final product (Fig. 2). This pathway was detected under both oxic conditions in the epilimnion and anoxic conditions in the hypolimnion, indicating its broad distribution across the stratified water column.

**Fig. 4.**
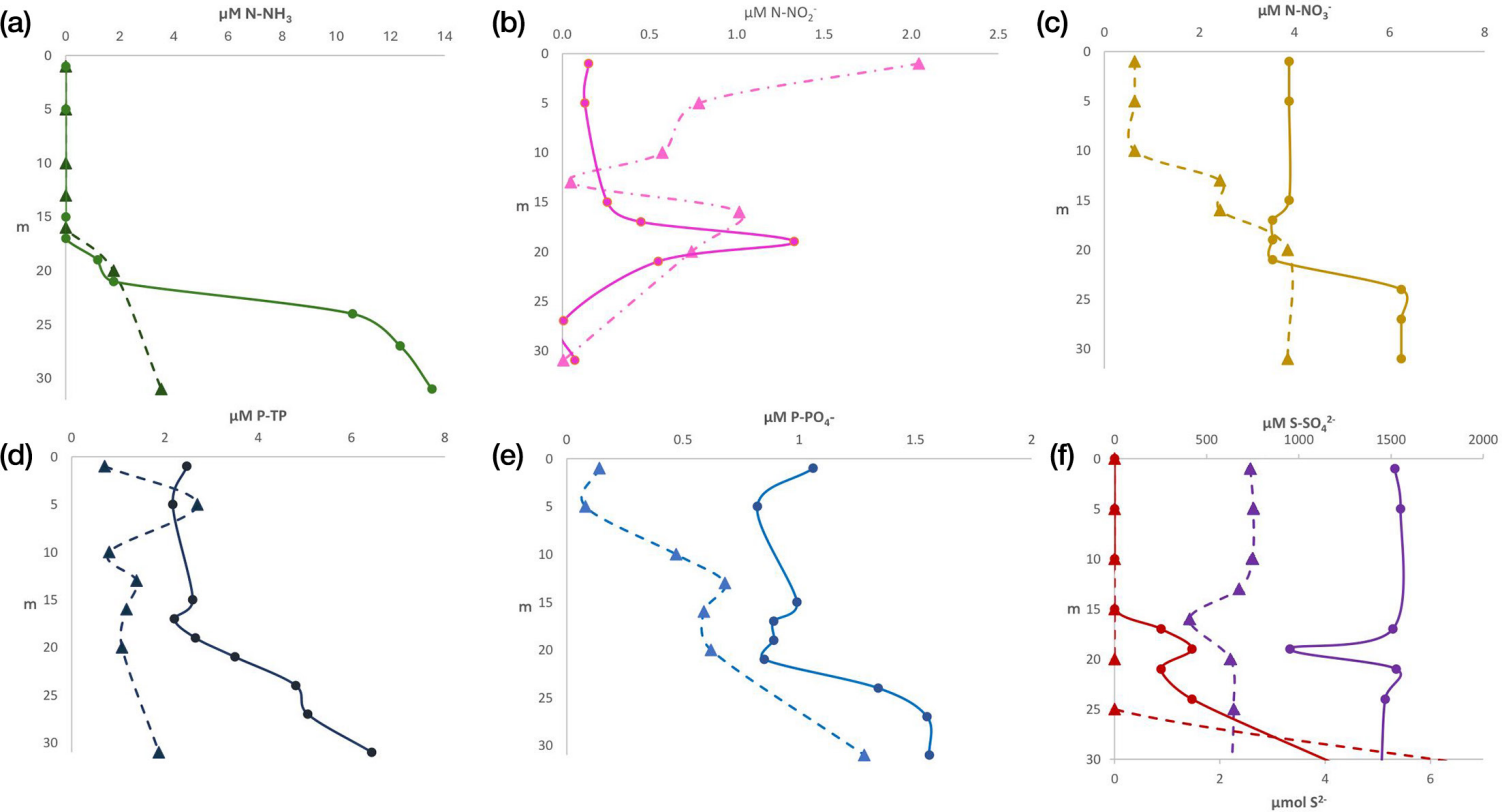
Vertical concentration profiles of nutrients and sulphur species in the water column. Panels show (**a**) ammonia (NH_3_), (**b**) nitrite (NO₂⁻), (**c**) nitrate (NO₃⁻), (**d**) total phosphorus (TP), (**e**) orthophosphate (PO₄³⁻) and (**f**) sulphur species. In panel f, sulphate (SO₄²⁻) is represented by purple lines and molecular sulphur (S⁰) by red lines. Dotted lines indicate early stratification, whereas solid lines indicate well-established stratification.

Acetate fermentation was annotated in several genomes with high coverage across all water-column layers during stratification (Fig. 2). Although this pathway is primarily anaerobic, it can also occur within the oxycline of the metalimnion. Accordingly, its prevalence increased progressively from the epilimnion towards the hypolimnion, where it was most prominent, reflecting enhanced anaerobic carbon processing under anoxic conditions (Fig. 3a.1, b.1). The widespread high concentrations of reactive soluble phosphorus observed throughout the lake (Fig. 4e) may further promote fermentative metabolism, given the close link between phosphorus availability and microbial organic carbon processing. Methanogenesis was not detected in the water column, including within the anoxic and chemically reduced hypolimnion (Fig. 2). This absence is consistent with the ORPs recorded during the study period, which remained above the thresholds typically required to sustain methanogenic activity (Fig. 3a.3, b.3).

Hydrogen generation from ethanol, a process rarely reported in natural aquatic systems, was detected in Lake Atexcac, with a higher number of genomes and greater coverage towards the reducing anoxic hypolimnion (Figs2  3a.1, b.1). In addition, fermentative hydrogen production was widespread throughout the water column and was more prevalent than ethanol-derived hydrogen, as indicated by higher genome numbers and coverages (Fig. 2). Together, these pathways contributed to hydrogen production across the stratified lake.

**Fig. 5.**
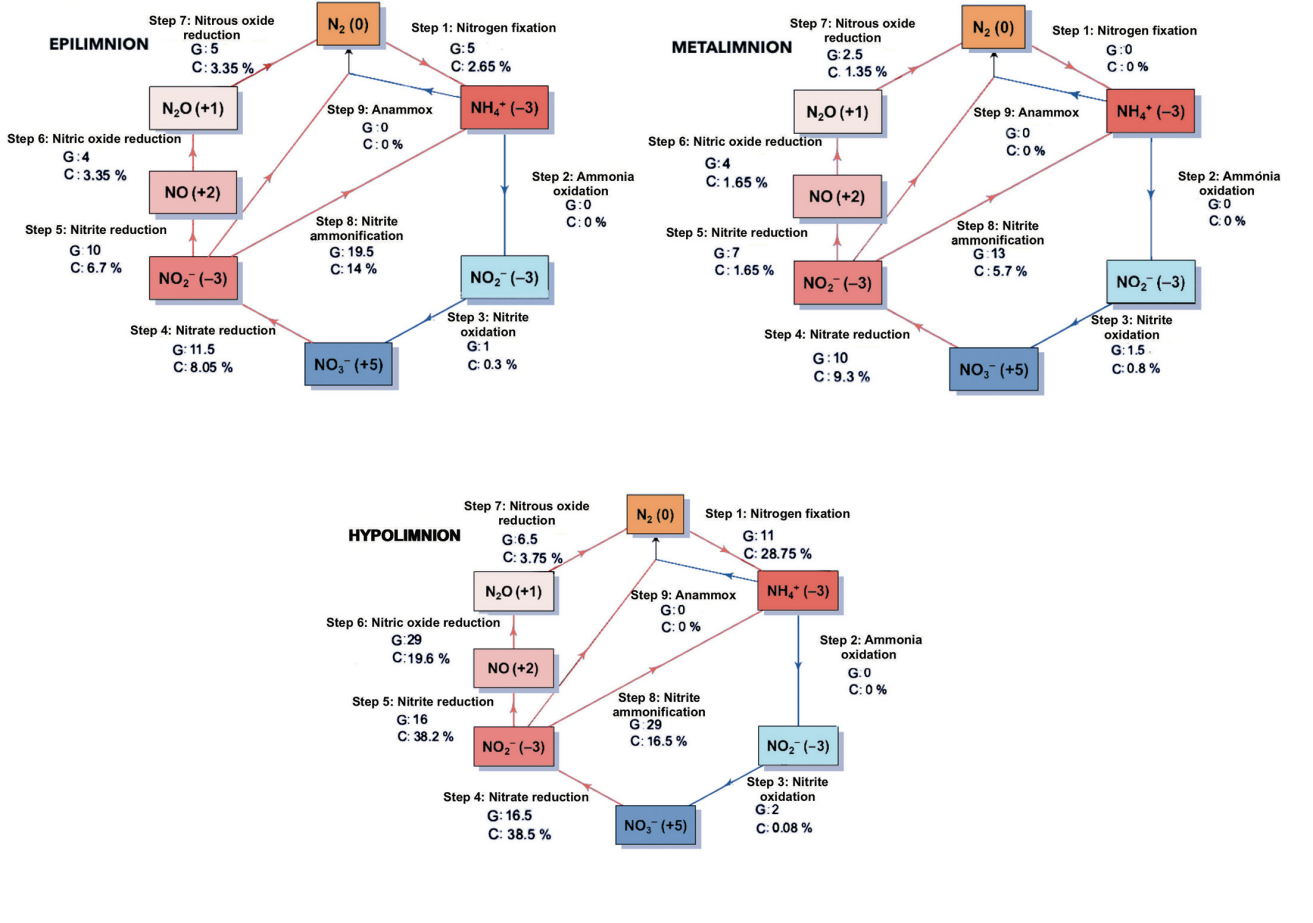
Mean number of genomes (G) and mean coverage (%) (C) of nitrogen cycle–related metabolic processes across water-column layers, averaged over early and well-established stratification periods. Red lines indicate reduction processes, whereas blue lines indicate oxidation processes.

Nitrogen fixation was detected in both the epilimnion and the hypolimnion; however, the highest number of genomes and greatest coverage occurred in the deep layer (Fig. 5). Nitrogen reduction reactions associated with denitrification also increased in representation towards the hypolimnion, although these processes were detected in fewer genomes and with lower coverage in the epilimnion and metalimnion.

Dissimilatory nitrate reduction to ammonium (DNRA) was an important nitrogen transformation in Lake Atexcac and was associated with the accumulation of ammonia along the water column, particularly towards deeper layers (Fig. 4a). Ammonia concentrations increased from the lower metalimnion into the hypolimnion, reaching their highest values in well-established stratification (Fig. 4a). The oxidation of ammonia to nitrite was not detected during the study period. Consistently, only a small number of genomes and low coverages were associated with nitrite oxidation to nitrate. In contrast, nitrate reduction to nitrite was represented by a higher number of genomes and greater coverage throughout the water column, highlighting the importance of nitrite as a central nitrogen intermediate. No genes associated with ANAMMOX were detected in either sampling month (Fig. 5).

**Fig. 6.**
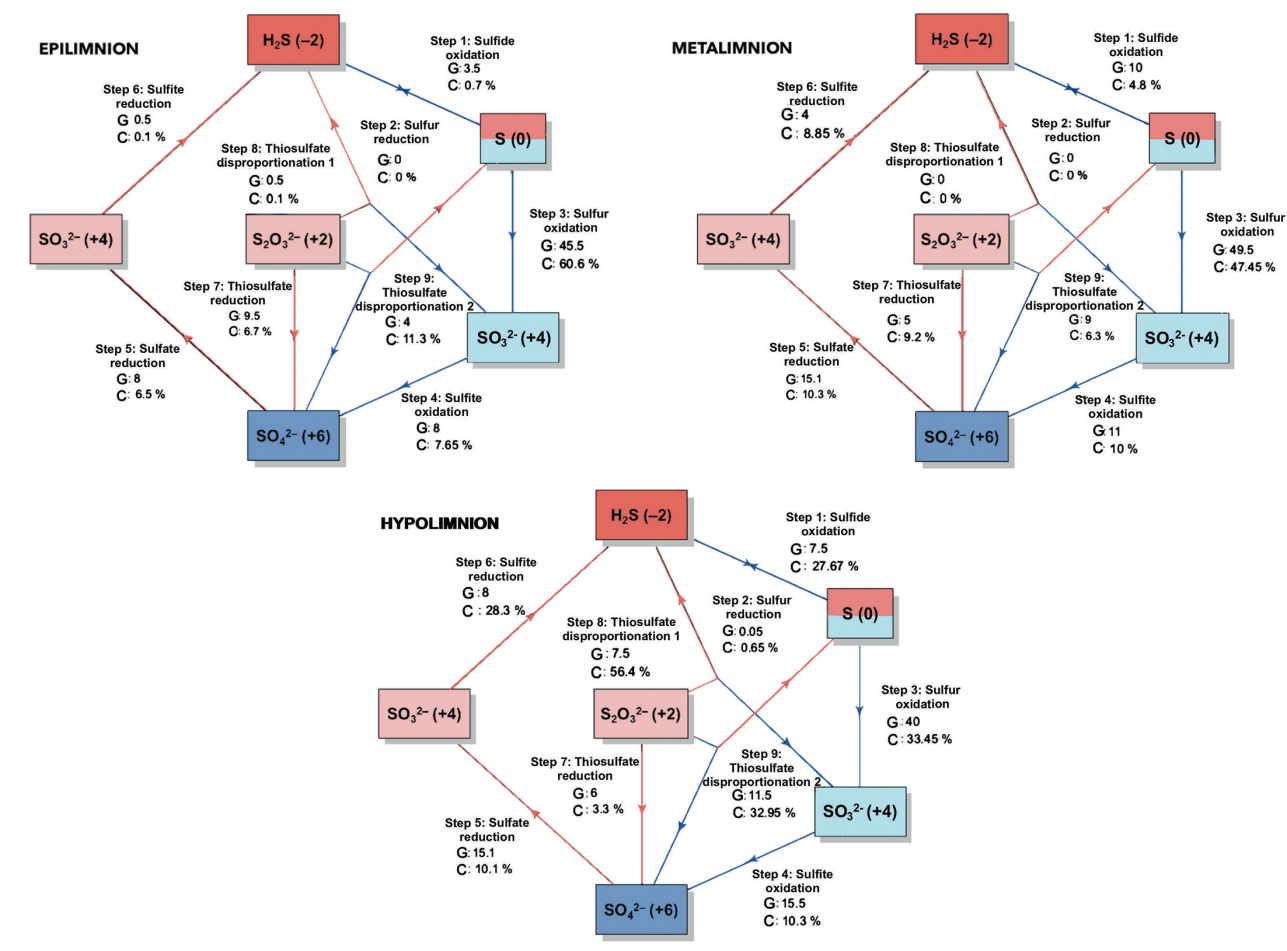
Mean number of genomes (G) and mean coverage (%) (C) of sulphur cycle–related metabolic processes across water-column layers, averaged over early and well-established stratification periods. Red lines indicate reduction processes, whereas blue lines indicate oxidation processes.

The sulphur cycle in Lake Atexcac exhibited high metabolic complexity, as evidenced by the diversity of genomes and gene coverages associated with sulphur oxidation and reduction processes across the water column (Fig. 6). Sulphur oxidation increased towards the hypolimnion as stratification progressed, coinciding with the establishment of anoxic conditions below 20 m and redox potentials below 0 mV (Fig. 3b.3). However, sulphur oxidation was also detected in the epilimnion, indicating that this process occurs under both oxic and anoxic conditions.

Two thiosulphate disproportionation pathways were identified: one producing the more reduced compound hydrogen sulphide and another yielding the more oxidized compound sulphite. The hydrogen sulphide-producing pathway was detected almost exclusively in the hypolimnion, whereas sulphite-producing disproportionation occurred across all three strata and increased from the epilimnion towards the hypolimnion based on genome abundance and coverage (Fig. 6). Sulphate reduction to hydrogen sulphide via sulphite was also detected throughout the water column but was most pronounced in the metalimnion and hypolimnion (Fig. 6). Overall, sulphate reduction was primarily associated with oxygen-deficient conditions, occurring mainly in the hypolimnion and secondarily in the oxycline of the metalimnion, although low genome numbers and coverages indicated limited activity in the epilimnion.

NMDS of MASH distances computed from quality-trimmed metagenomic reads is shown in Fig. S2. The ordination had a stress value of 0.154 with two dimensions, indicating an acceptable fit. Epilimnion and hypolimnion samples clustered primarily by hydrological phase, whereas metalimnion samples were consistently the most distinct across both the stratification and whiting event datasets.

### Microbial community composition

**Fig. 7.**
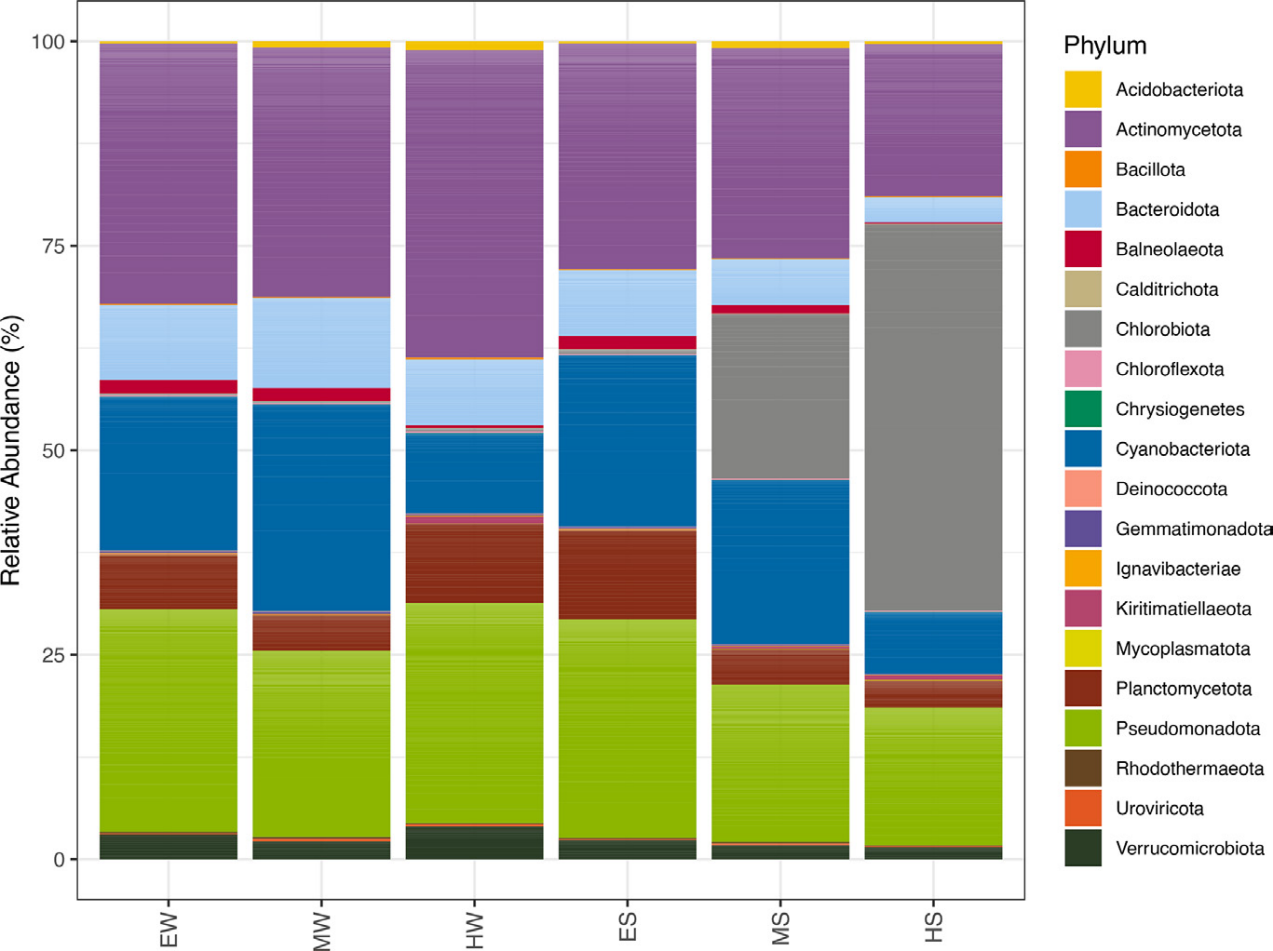
Read relative abundance of different taxonomic groups based on Kaiju assignment. Unclassified reads that correspond to phyla <0.01% of the abundance are not shown. The six samples analysed are represented as follows: EW, epilimnion whiting event; MW, metalimnion whiting event; HW, hypolimnion whiting event; ES, epilimnion stratification; MS, metalimnion stratification; HS, hypolimnion stratification.

In total, 20 taxa had genetic abundances above the defined threshold of the total reads according to Kaiju (i.e. >0.01% of the abundance). Different phyla were found in all samples with higher abundances, namely *Actinomycetota*, *Bacteroidota*, *Cyanobacteriota*, *Planctomycetota*, *Pseudomonadota* and *Verrucomicrobiota* (Fig. 7). Samples from the metalimnion and hypolimnion layers of the stratified water column exhibited a high relative abundance of *Chlorobiota* reads, accounting for 20.1 and 47.2% of the total sequences, respectively. *Chlorobiota* was the most dominant phylum in the hypolimnion under well-established stratification conditions (HS) (Fig. 7). A deeper inspection of the *Chlorobiota* reads for the HS sample showed that 48% of the *Chlorobiota* reads corresponded to *Chlorobium limnicola* DSM 254, while in the MS sample, this same species accounted for 30% of the *Chlorobiota* reads.

In both the MS and HS samples, only one MAG belonging to the genus *Chlorobium* was found. In the former, the MAG contained genes for thiosulphate disproportionation (to hydrogen sulphide and sulphite), sulphite reduction and sulphide oxidation to elemental sulphur (Fig. S3), while in the latter, it had the same genes for this cycle and another mechanism for thiosulphate disproportionation (to elemental sulphur and sulphate) (Fig. S4). These genomes were classified as medium-quality MAGs.

For *Pseudomonadota*, the most abundant class for both periods in the epilimnion and metalimnion was *Alphaproteobacteria*. Noteworthy, for this class, the most abundant species was *Candidatus Fonsibacter ubiquis*, which comprised between 6 and 18% of the reads for this phylum. For the hypolimnion, *Betaproteobacteria* comprised most of the *Pseudomonadota* reads. *Actinomycetota* reads were highly abundant in all samples (18–37%). A deeper inspection of the reads showed that in all samples, the *Corynebacteriales* order represented the highest proportion of reads, except for the sample corresponding to the epilimnion during stratification, where the *Micrococcales* order was dominant. Noteworthy, for both periods, the proportion of *Corynebacteriales* increased considerably in the hypolimnion (73 and 78%) compared to the epilimnion and metalimnion samples (25–47%).

For *Bacteroidota* in the epilimnion and metalimnion, the *Flavobacteriales* clade was the most dominant (48–65%) for both periods, followed by *Cytophagales* (16–22%). For the hypolimnion during the whiting event, the proportion of *Flavobacteriales* decreased (36%), while *Bacteroidia* was the second most represented (32%). In the HS sample, a similar pattern was found, with most reads being assigned to *Bacteroidia* (33%) followed by *Flavobacteriales* (29%). The *Planctomycetota* phylum reads ranged between 3.8 and 10.8%. For all samples, most reads from this phylum were annotated as belonging to *Pirellulales* followed by *Planctomycetaceae*. The only exception to this read classification pattern in *Planctomycetota* was the hypolimnion during the whiting event, where the reverse pattern was observed. *Verrucomicrobiota* were present in every sample in smaller proportions (1.5–4%), and most reads were assigned to *Opitutae* followed by *Verrucomicrobiales*. Differences in heterotrophic bacterial communities were most pronounced in the hypolimnion. Analysis of carbohydrate utilization genes inferred using METABOLIC [42] indicated an increased number of genomes encoding enzymes involved in the stepwise degradation of structurally complex polysaccharides. These pathways include the enzymatic breakdown of alginate, arabinogalactan, cellulose, xylan and galacturonide polymers, reflecting enhanced microbial capacity for the depolymerization and processing of high-molecular-weight carbohydrates under hypolimnetic conditions (Fig. S5).

From all the ‘cellular organism’ reads, a very small fraction corresponded to *Archaea* (0.2–0.3%) for all samples, with most of these reads belonging to *Euryarchaeota*. This group contained reads for *Methanomicrobia* and halophilic clades. From all the samples, only one medium-quality archaeal genome was found in the hypolimnion whiting event sample that belonged to the *Pacearchaeales* order. *Cyanobacteriota* composed a considerable fraction of the reads in the epilimnion and metalimnion for both periods, and the *Synechococcaceae* accounted for most of the reads for this phylum in all samples (63–74%), followed by *Prochloraceae* (16–22%). All cyanobacterial MAGs obtained also belonged to these families. Other clades accounted for ~1–2%, including *Pseudanabaenales*, *Nostocales* and *Oscillatoriales*. In all samples, between 0.2 and 0.5% of the cyanobacterial reads corresponded to *Gloeobacteraceae*.

In the read-based analysis, a small fraction of viral reads was detected in all samples (0.2–0.7% of the total reads), with bacteriophages of the family *Myoviridae* being the most represented in all samples. A deeper inspection of this family showed that during the stratification period, in the metalimnion and hypolimnion samples, most reads mapped to *Synechococcus* phages, while for the rest of the samples, most mapped to a *Flavobacterium* phage.

## Discussion

Across biogeochemical pathways, Lake Atexcac shows strong vertical structuring consistent with persistent stratification. The bacterial community was dominated by *Actinomycetota*, *Bacteroidota*, *Planctomycetota* and *Verrucomicrobiota*, consistent with other maar lakes by Iniesto *et al.* [45], suggesting that stratification and steep vertical gradients act as ecological filters that repeatedly select for similar communities. This taxonomic structure aligns with complementary roles in carbon fixation near the surface and organic matter remineralization and redox-coupled cycling at depth.

The dynamics of carbon fixation in the epilimnion and metalimnion of Lake Atexcac reflect characteristic features of saline, oligotrophic maar lakes, where picocyanobacteria such as *Synechococcus* often dominate primary production. Cyanobacterial reads were mainly picocyanobacteria with dominance of the order *Synechococcaceae*, followed by *Prochloraceae*, the latter being described for the first time in Lake Atexcac. Filamentous taxa were less abundant, matching regional succession patterns [46,47]. During the early stratification period, their capacity to utilize bicarbonate and induce calcium carbonate precipitation was observed, in agreement with previous reports describing their role in whiting events [7,48]. Their abundance in tropical, low-productivity systems [22], together with their ability to photosynthesize at extremely low photosynthetically active radiation (PAR) levels down to ~0.1% of surface irradiance [49] helps explain the formation of DCM and the persistence of primary productivity at depth. The presence of phycoerythrin-rich *Synechococcus*, capable of absorbing blue light (~460 nm) through a phycobilisome system containing phycoerythrin fluorochromes, further extends the depth of primary productivity in oligotrophic systems, as these wavelengths penetrate to greater depths [50]. Accordingly, phycoerythrin-rich *Synechococcus*, such as those recorded in Lake Atexcac, tend to dominate under conditions where the vertical attenuation coefficient of PAR is lower than 0.55 m⁻¹, and green–blue wavelengths (480–500 nm) remain available [51]. In addition, anoxygenic photosynthetic bacteria affiliated with *Alpha*- and *Gammaproteobacteria* have been reported in these lakes [45] and may contribute to enhanced carbon fixation rates in the anoxic hypolimnion, as proposed by Havas *et al.* [52]. These bacteria are well adapted to extremely low light intensities, even below 5×10⁻⁴% of surface irradiance [53].

Organic carbon oxidation in Lake Atexcac is primarily driven by the high availability of dissolved organic carbon (DOC), which constitutes nearly 98% of the total organic carbon pool and increases from the epilimnion towards the metalimnion and hypolimnion. This vertical DOC gradient sustains intense aerobic heterotrophic respiration and contributes to progressive oxygen depletion with depth [52]. The dissolved carbon pool fuels abundant heterotrophic bacterial communities, with average densities of 1.49–1.98×10⁶ cells ml⁻¹ during early stratification and 2.15–2.44×10⁶ cells ml⁻¹ under well-established stratification, reaching maximum values in the metalimnion [54]. Community composition and functional potential both point to heterotrophic processing of organic matter, with polysaccharide-degrading lineages such as *Bacteroidota*, *Planctomycetota* and *Verrucomicrobiota* [55,56]. In this context, the enrichment of carbohydrate-active enzymes in the hypolimnion during the whiting event is consistent with slow degradation of sinking particles (‘lake snow’) produced by earlier blooms, providing sustained substrates for deeper heterotrophy.

Acetate oxidation in Lake Atexcac occurs across redox gradients, being driven by aerobic heterotrophs in the oxic epilimnion and by anaerobic pathways in the anoxic hypolimnion, including syntrophic acetate oxidation coupled to hydrogen or formate transfer and alternative electron acceptors [57]. This dual occurrence highlights acetate as a central metabolic intermediate that links fermentative processes with both aerobic respiration and anaerobic carbon cycling throughout the stratified water column. In this context, acetate fermentation has been reported to occur predominantly in the hypolimnion, where bacteria extract energy from complex organic substrates, particularly under conditions of elevated reactive soluble phosphorus, further reinforcing the role of acetate as a key node connecting organic matter degradation and carbon turnover in deeper waters [58].

The absence of methanogenesis in the water column is consistent with the prevailing redox conditions during stratification, as hypolimnetic ORP values around −100 mV are insufficient to sustain methanogenic activity, which typically requires strongly reducing conditions (≤−200 to −300 mV) following the depletion of alternative electron acceptors [59]. Although acetate is a key methanogenic substrate, acetate-driven methanogenesis is generally restricted to sediments where redox conditions are more reduced and stable, suggesting that methane production in Lake Atexcac is confined to benthic environments rather than the water column [60].

Hydrogen metabolism represents a key energetic pathway in aquatic microbial communities, supporting diverse aerobic and anaerobic strategies through the activity of iron-containing hydrogenases that mediate H₂-based respiration, fermentation and carbon fixation [61]. In Lake Atexcac, these processes are likely widespread across the stratified water column and may be further enhanced by the high iron availability associated with the magmatic origin of the system [62], which supports the synthesis and function of iron-dependent hydrogenases.

In Lake Atexcac, surface-water nitrogen fixation during early stratification has been previously attributed to the heterocystous cyanobacterium *Nodularia cf. spumigena*, typical of hyposaline lakes [63]. Its absence during the late early stratification and well-established stratification periods sampled in this study indicates a seasonal decline in abundance and suggests a reduced contribution of cyanobacterial nitrogen fixation at the time of sampling. Under these conditions, nitrogen fixation appears to shift towards deeper layers of the water column, where the establishment of microaerobic to anaerobic conditions in the hypolimnion favours diazotrophy by non-heterocystous cyanobacteria and diverse heterotrophic micro-organisms [64]. Stratified, low-oxygen water columns commonly harbour the genetic potential for active N₂ fixation, as evidenced by the widespread presence of nifH genes [65], with increased abundances reported along vertical redox gradients in subtropical reservoirs [16]. Consistently, studies in tropical Lake Tanganyika have identified diverse diazotrophic taxa at suboxic and anoxic depths, supporting the role of deep, low-oxygen layers as key niches for nitrogen fixation in stratified lakes [15].

The presence of genomes associated with denitrification in the oxic epilimnion of Lake Atexcac suggests that this process may occur under aerobic conditions. Aerobic denitrification has been reported in a wide range of natural and artificial aquatic systems and is commonly associated with members of the *α*-, *β*- and *γ*-*Proteobacteria* [66]. In contrast, canonical denitrification, which operates as an anaerobic respiratory process, appears to prevail in the metalimnion and hypolimnion. In maar-type lakes, high abundances of denitrification genes have been consistently reported in the oxycline and hypolimnetic layers, highlighting the importance of redox gradients in structuring nitrogen removal pathways [18].

DNRA in Lake Atexcac is relevant for the accumulation of ammonia along the water column, being higher towards the deep layer, particularly in the hypolimnion. In fact, an increase of ammonia is observed from the bottom of the metalimnion towards the hypolimnion, with a higher concentration in the well-established stratification. Similarly, anaerobic denitrification has been recorded in maar Lake Alchichica [18] and in tropical Lake Yojoa, Honduras, which also presents an anoxic condition [20].

The absence of ammonia oxidation in Lake Atexcac is consistent with observations from other warm monomictic systems, such as Lake Yojoa, where *amoA* expression was not detected [20], although this gene has been reported in the oxycline of the hyposaline Lake Alchichica [18] indicating system-specific controls on nitrification. The relatively low ammonia concentrations in the hypolimnion of Lake Atexcac compared to Lake Yojoa may further limit the activity of ammonia-oxidizing archaea and bacteria [67]. The dominance of nitrate reduction over nitrite oxidation suggests that nitrite functions as a key intermediate supporting denitrification and DNRA processes. Additionally, the absence of ANAMMOX genes is consistent with previous studies indicating that this process requires strictly anoxic and strongly reducing conditions, which are more commonly found in sediments than in the water column.

The increase in sulphur oxidation towards the hypolimnion under anoxic conditions suggests the involvement of phototrophic sulphur-oxidizing bacteria, such as green sulphur bacteria (*Chlorobiaceae*) and purple sulphur bacteria (*Chromatiaceae*), which perform anoxygenic photosynthesis in low-light environments [68]. Similar taxa, including members of the orders *Chromatiales* and *Ectothiorhodospirales*, have been reported in the hyposaline maar Lake Alchichica, particularly genera such as *Thiocapsa* and *Thioalkalivibrio*, which are abundant during stratification under anoxic conditions where PAR exceeds 0.1% [69].

The presence of *Chlorobiota* in lakes and other environmental systems is traditionally linked to the sulphur cycle. In this study, *Chlorobiota* was particularly abundant in deeper stratified layers, consistent with its association with sulphur-linked metabolisms. The phylum has not been widely reported in lake systems using 16S amplicon sequencing approaches, probably because it has been reported as *Bacteroidota*, with which it shares a close evolutionary relationship [70]. The sequenced genomes of *C. limnicola* show that specific strains of this species have plasmids that harbour genes for the disproportionation of thiosulphate [71]. Thiosulphate oxidation in *Chlorobium limnicola* is metabolized by enzymes coded in a biosynthetic gene cluster of seven genes that are linked to a periplasmic complex [72]. Analyses of *Chlorobiota* genomes in different stratified lakes have shown that SoxB genes that oxidize thiosulphate via a thiosulfohydrolase are not widespread in this clade and were likely lost in most species [73]. In line with these genome-based observations, the *Chlorobium* MAGs recovered here contained genes associated with thiosulphate disproportionation and related sulphur transformations. These results offer a novel insight into specific pathways of relevance for the sulphur cycle in maar lakes.

Sulphur oxidation under oxic conditions in the epilimnion is likely mediated by obligate chemolithoautotrophic bacteria, such as *Thioalkalivibrio*, which have been widely reported in sodic lakes [74]. More broadly, chemolithotrophic sulphur-oxidizing bacteria belonging to *Alphaproteobacteria*, *Gammaproteobacteria* and *Epsilonproteobacteria* have been documented in comparable aquatic environments [75], supporting the metabolic versatility of sulphur oxidation across redox gradients in stratified systems.

Thiosulphate oxidation involves genes that are widely distributed across diverse prokaryotic lineages, including phyla such as *Parcubacteria*, *Actinomycetota*, *Chloroflexota*, *Bacteroidota* and, within the phylum *Pseudomonadota*, the classes *Alphaproteobacteria*, *Gammaproteobacteria* and *Deltaproteobacteria* [76].

In Lake Atexcac, this metabolic diversity co-occurs with an active sulphur-reducing community, as sulphate-reducing bacteria have been detected at abundances on the order of 10³ cells ml⁻¹ in aerobic and microaerobic zones and up to 10⁴ cells ml⁻¹ in anaerobic waters. During whiting events, similarly high abundances have been reported in the microaerobic and anaerobic zones using immunoassay-based detection [77]. This vertical distribution is consistent with patterns observed for sulphate-reducing bacteria in the nearby Lake Alchichica using FISH analyses [3]. The occurrence of sulphate reduction in the oxic epilimnion may be explained by the presence of oxygen-tolerant sulphate-reducing bacteria [78]. In Lake Atexcac, phenotypic characterization has suggested the presence of four SRB genera – *Desulfovibrio*, *Desulfotomaculum*, *Desulfobulbus* and *Desulfobacter* – highlighting the coexistence of sulphur oxidation and reduction pathways across the lake’s redox gradient [77].

*Archaea* represented only a small fraction of reads and were dominated by *Euryarchaeota*; recovery of a *Pacearchaeales* MAG from deep water is consistent with oligotrophic lake observations [79] and with expected niches in a hyposaline, stratified system. Finally, given the filtration size fraction, many viral sequences likely correspond to prophages within host genomes; this is relevant because cyanophages often carry auxiliary metabolic genes that can influence host metabolism and nutrient cycling [80,81].

The pronounced distinctiveness of the metalimnion displayed in the NMDS suggests increased genetic turnover across the thermocline, which may reflect stronger microhabitat heterogeneity and niche partitioning associated with steep physicochemical gradients [82,83]. Similar stratification-driven divergence of microbial communities has been reported in other lake systems, including a eutrophic seepage lake and the dimictic Tibetan Lake Nam Co, where the epilimnion and metalimnion often exhibit the highest community heterogeneity [84,85]. The NDMS shows a high genetic dissimilarity in the metalimnion that can be associated with a higher bacterial species number, a pattern that may reflect the high micro-habitat heterogeneity and niche partitioning across the thermocline. During stratification, the metalimnion also yielded the most MAGs, whereas during the whiting event, most MAGs were recovered from the hypolimnion (Table 1).

Notably, metagenome- and MAG-based annotations reflect functional potential rather than confirmed *in situ* activity, and for this reason, the pathway-level interpretations should be viewed as hypotheses. Consistent with this, studies integrating matched metagenomes with metatranscriptomes and metaproteomes show that DNA-level gene abundance can diverge from transcription and protein detection across taxa and functions [86,88]. Therefore, a key limitation is that the functional conclusions presented in this study are based on DNA sequence data, and validating pathway activity will require complementary activity-linked measurements.

## Conclusions

Thermal stratification and the resulting redox gradients emerge as the main controls of microbial activity in Lake Atexcac, shaping the vertical organization of carbon, nitrogen and sulphur metabolisms and allowing the simultaneous operation of aerobic and anaerobic pathways within the water column. Carbon cycling is largely driven by DOC and metabolically versatile micro-organisms, with acetate and hydrogen functioning as central intermediates that link fermentation, respiration and anaerobic oxidation processes, while methanogenesis is excluded from the water column due to the absence of sufficiently reducing conditions. Nitrogen transformations are dominated by reductive pathways under low-oxygen conditions, where nitrogen fixation, denitrification and DNRA prevail, promoting ammonia accumulation and internal nitrogen retention, whereas nitrification played only a minor role and anammox was not recorded. Sulphur cycling displays high metabolic complexity and is tightly coupled to carbon and nitrogen processes, as sulphur oxidation and sulphate reduction co-occur along redox gradients, underscoring the central role of sulphur metabolism in sustaining anaerobic energy flow in the lake.

This study represents, to our knowledge, the first application of shotgun metagenomics to resolve the temporal and vertical dynamics of microbial communities and their biogeochemical potential in a maar lake, and the first to describe microbial community structure and metabolic potential during a whiting event in any lake. The use of deep sequencing (~100 million reads per sample) enabled the recovery of a broad diversity of MAGs, providing a robust framework for future comparative analyses with other saline lake microbial genomes. In particular, the importance of the phylum *Chlorobiota* in the sulphur cycle was revealed, a group that had not been previously detected using amplicon-based approaches. MAGs affiliated with the genus *Chlorobium* corresponded to distinct ecotypes with differentiated genetic repertoires for thiosulphate disproportionation, suggesting niche partitioning within the water column. The detection of multiple previously described taxa and similarities with other lacustrine systems further contextualize Lake Atexcac within broader aquatic microbial frameworks, while the absence of anammox in the water column points to its restriction to sediments and highlights the need to integrate sediment core sampling with water-column metagenomics. Overall, the large number of recovered MAGs and the metabolic complexity of the microbial communities emphasize the intricate interactions underpinning biogeochemical cycles in this system and underscore the need for complementary molecular approaches, as well as targeted isolation and cultivation of key taxa guided by MAG-inferred metabolisms.

Overall, our results provide a transferable framework for future microbial and biogeochemical surveys in stratified aquatic systems, highlighting the value of pairing depth-resolved sampling across redox gradients with deep shotgun metagenomics to link community turnover to process potential. Applied to other maar lakes, as well as larger freshwater lakes and reservoirs, this approach can help identify when and where key transformations intensify during stratification and guide the design of monitoring programs and comparative studies across aquatic bodies.

## Supplementary material

10.1099/mic.0.001714 Supplementary Material 1.

## Abbreviations

anammox: anaerobic ammonium oxidation
APP: autotrophic picoplankton
DCM: deep chlorophyll maxima
DNRA: dissimilatory nitrate reduction to ammonium
DOC: dissolved organic carbon
MAGs: metagenome-assembled genomes
NMDS: non-metric multidimensional scaling
ORP: oxidation–reduction potential
PAR: photosynthetically active radiation
qPCR: quantitative PCR
