# Supplementary material for: Metagenomic insight into the diversity and biogeochemical functions of microbial communities in the maar tropical Lake Atexcac
Source: Microbiology (Reading). 2026 Jun 4;172(6):001714. doi: 10.1099/mic.0.001714 (PMC13235802; doi:10.1099/mic.0.001714)
Supplement: Supplementary Material 1. [file mic-172-01714-s001.pdf]

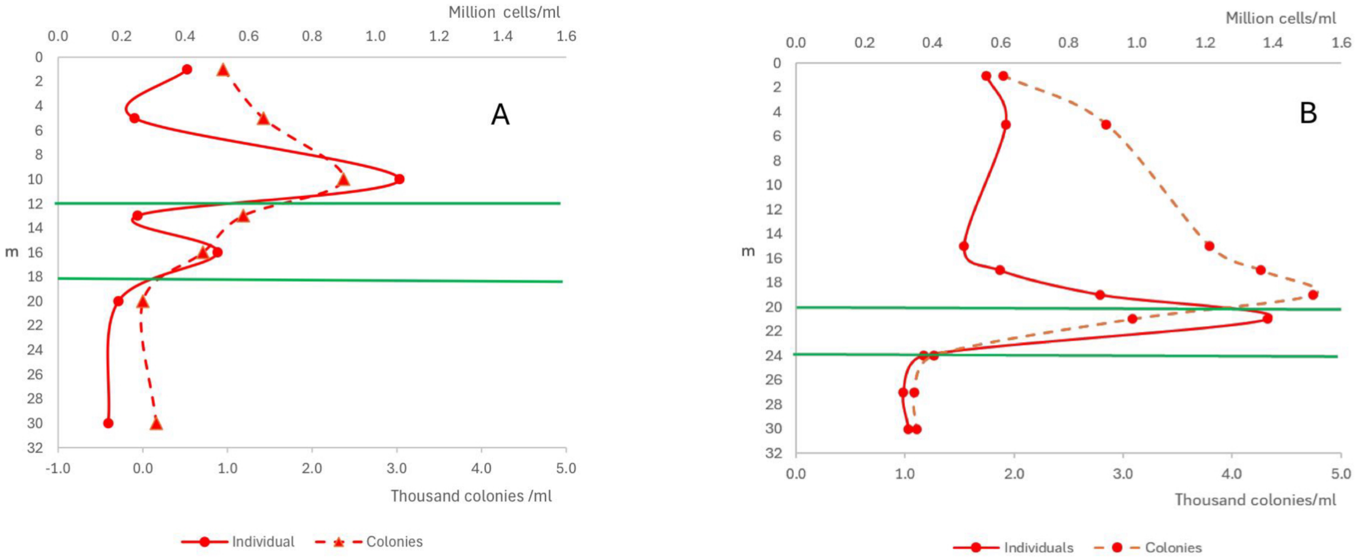

Sup. Fig. 1 Abundances of individual picocyanobacteria cells and colonies during the early- stratification (A) and well-established stratification (B).

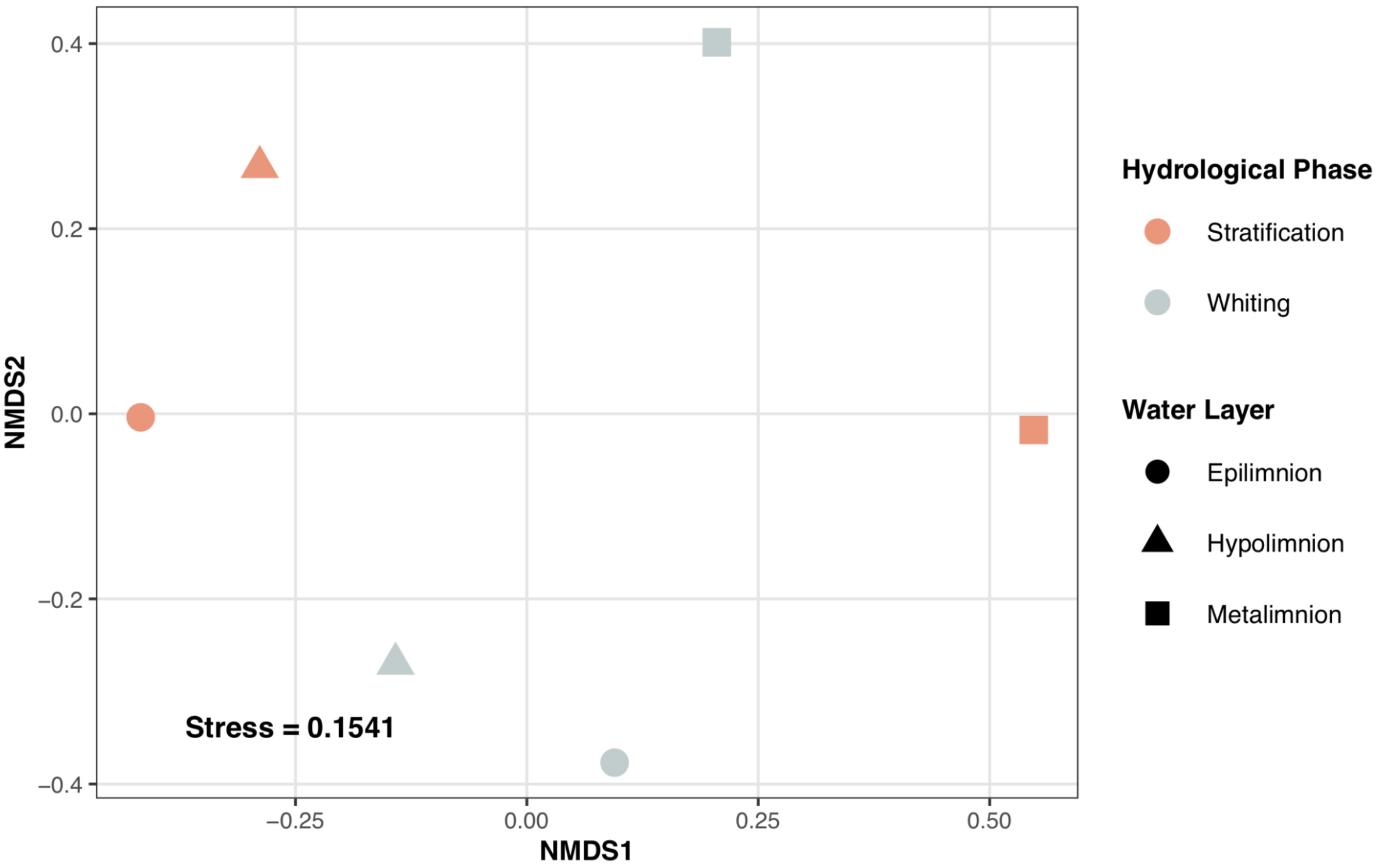

Sup. Fig. 2 Non-metric multidimensional scaling from the MASH distance of different sample metagenomes. Samples are colored by hydrological phase with the early stratification in the early stratification (Whiting) and well-established stratification (Stratification).

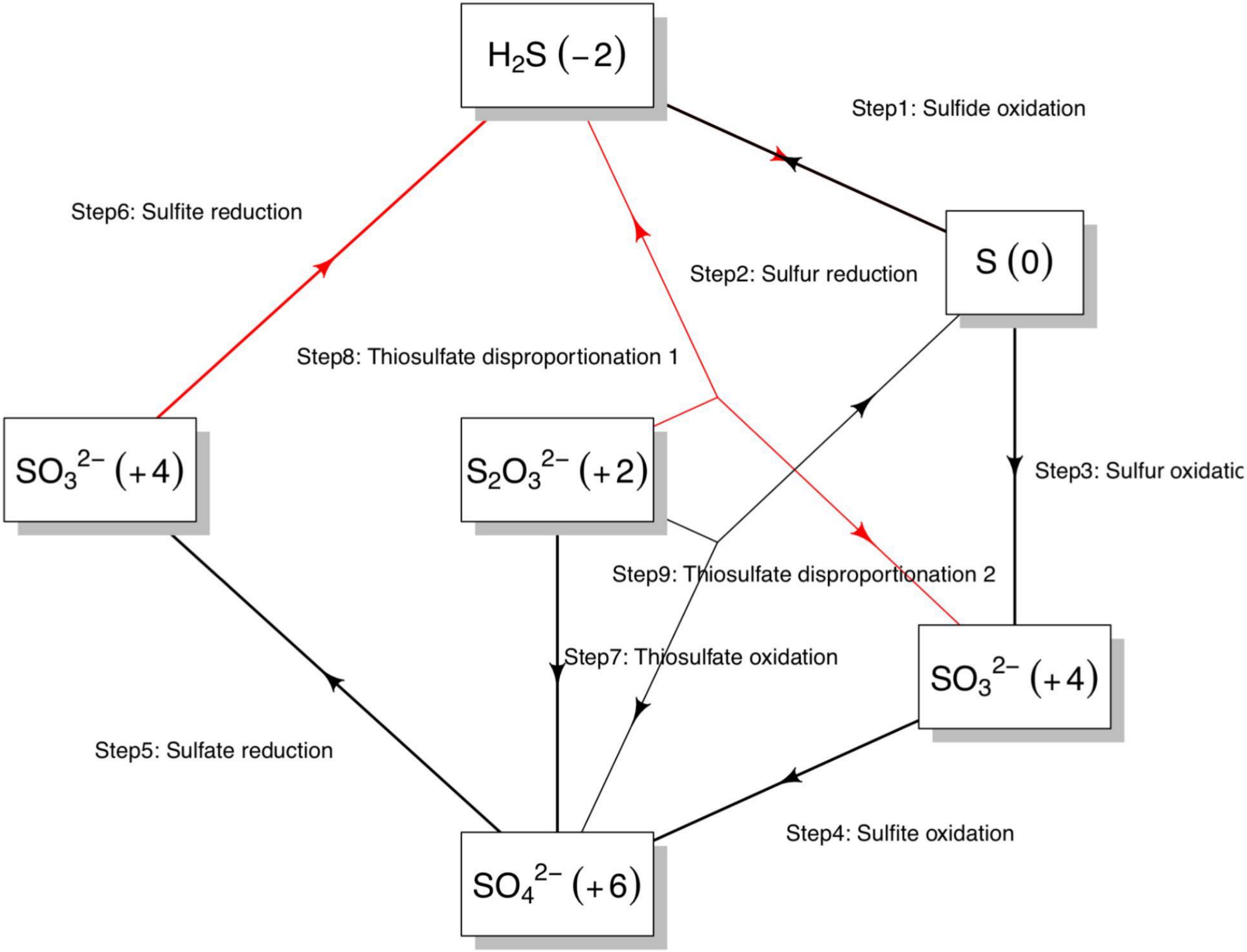

Sup. Fig. 3. Sulfur-cycle genes identified in a *Chlorobium* metagenome-assembled genome (MAG) recovered from the metalimnion sample during the well-established stratification period. Red arrows indicate reactions supported by the presence of the corresponding genes.

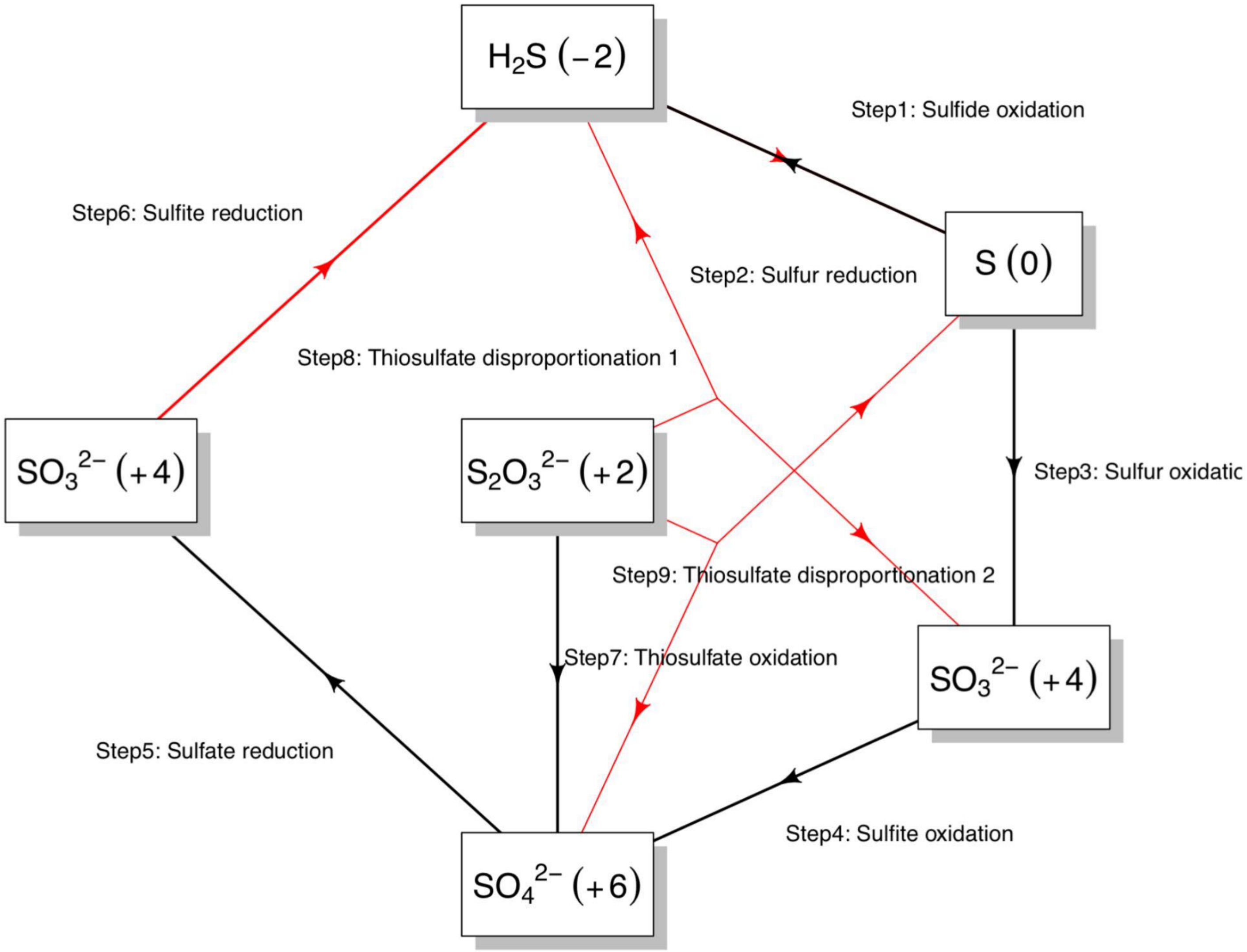

Sup. Fig. 4. Sulfur-cycle genes identified in a *Chlorobium* metagenome-assembled genome (MAG) recovered from the hypolimnion sample during the well-established stratification period. Red arrows indicate reactions supported by the presence of the corresponding genes.

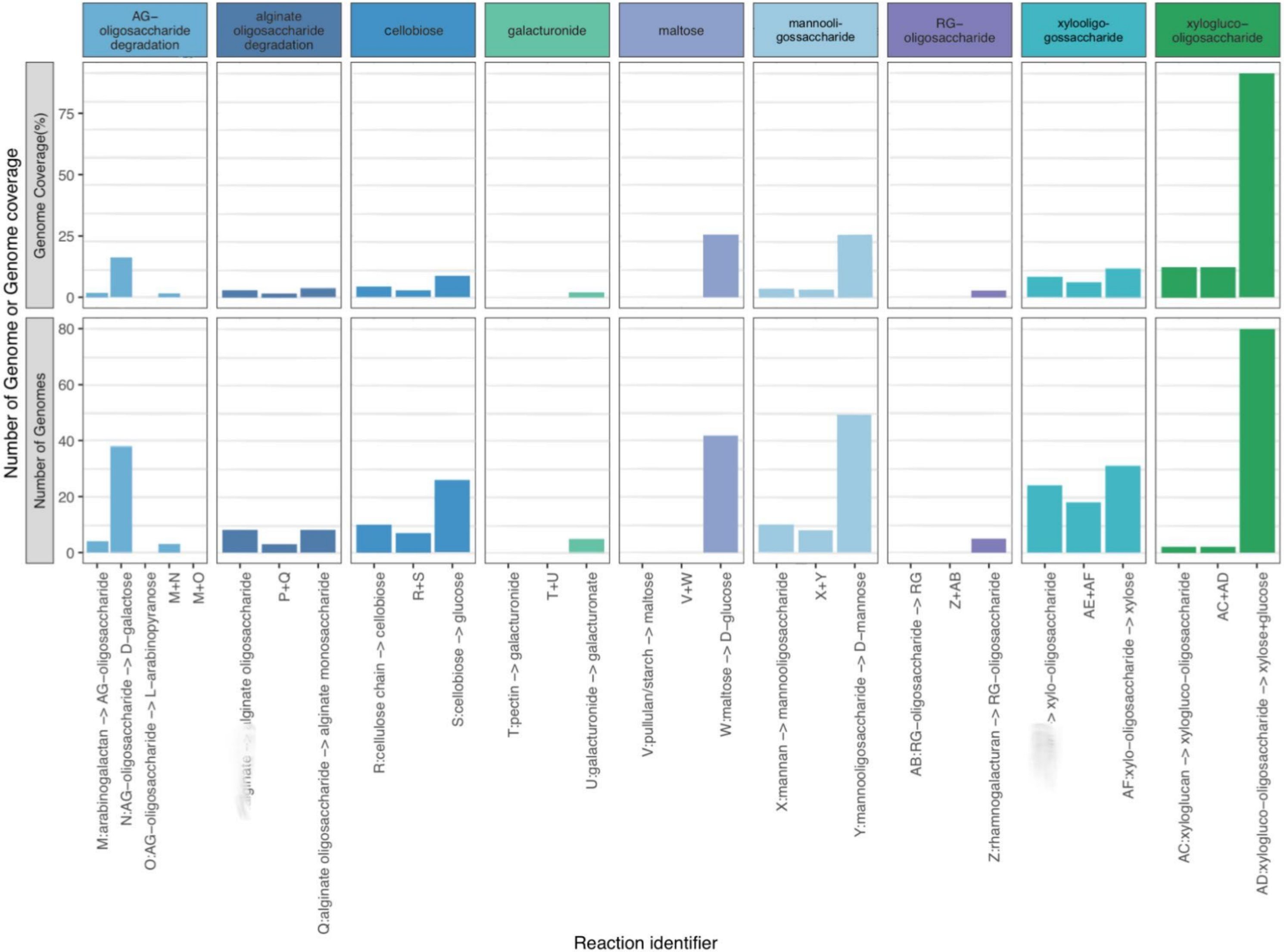

Sup. Fig. 5. Sequential plots of complex polysaccharide degradation genes in the sample corresponding to the hypolimnion in the whiting event. Upper plots show genome coverage, while lower plots show the total number of genomes in the samples.
